# Neuregulin 1 improves complex 2-mediated mitochondrial respiration in skeletal muscle of healthy and diabetic mice

**DOI:** 10.1038/s41598-017-02029-z

**Published:** 2017-05-11

**Authors:** Gaël Ennequin, Frederic Capel, Kevin Caillaud, Vivien Chavanelle, Monique Etienne, Allison Teixeira, Xinyan Li, Nathalie Boisseau, Pascal Sirvent

**Affiliations:** 1 Université Clermont Auvergne, Laboratoire des Adaptations Métaboliques à l’Exercice en Conditions Physiologiques et Pathologiques (AME2P), F-63000 Clermont-Ferrand, France; 2 INRA UMR1019 Nutrition Humaine, Laboratoire de Nutrition Humaine, Université d’Auvergne, CRNH, 58 rue Montalembert BP321, 63009 Clermont Ferrand, CEDEX 1 France; 3 Zensun Sci & Tech Ltd., Shanghai, China; 4 PEPITE EA4267 and Exercise Performance Health Innovation Platform University, Bourgogne Franche-Comté, F-25000 Besançon France

## Abstract

It has been reported that neuregulin1 (NRG1) improves glucose tolerance in healthy and diabetic rodents. *In vitro* studies also suggest that NRG1 regulates myocyte oxidative capacity. To confirm this observation *in vivo*, we evaluated the effect on mitochondrial function of an 8-week treatment with NRG1 in db/db diabetic mice and C57BL/6JRJ healthy controls. NRG1 treatment improved complex 2-mediated mitochondrial respiration in the *gastrocnemius* of both control and diabetic mice and increased mitochondrial complex 2 subunit content by 2-fold. This effect was not associated with an increase in mitochondrial biogenesis markers. Enhanced ERBB4 phosphorylation could mediate NRG1 effects on mitochondrial function through signalling pathways, independently of ERK1/2, AKT or AMPK.

## Introduction

Neuregulin 1 (NRG1) is a cytokine that belongs to a family of proteins structurally related to epidermal growth factors (EGF)^1^. NRG1 binds to the erythroblastic leukaemia viral oncogene homologues 3 and 4 (ERBB3 and 4) receptors through its EGF-like domain to activate various downstream signalling events^2, 3^. It has been shown that NRG1 plays a key role in myogenesis^4^, acetylcholine receptor formation^5^, muscle fibre survival^6^ and muscle regeneration^7^.

More recently, we have reported that endurance exercise training activates the NRG1/ERBB4 pathway in skeletal muscle of obeserats^8^. However, the underlying physiological consequences are unknown. Interestingly, previous studies suggest that NRG1 might be involved in the regulation of muscle energy metabolism^9^, possibly in part via an effect on mitochondrial biogenesis and function, a well-known target of exercise training. Indeed, it was shown that 48 h-incubation with NRG1 increases the oxidative capacity and the expression of mitochondrial-specific genes in L6E9 and C2C12 muscle cells. These effects are mediated by the peroxisome proliferator-activated receptor beta (PPARβ) and PPAR-gamma coactivator 1-alpha (PGC-1α) signalling pathway^10^. Experiments in other cell types confirmed that the NRG1/ERBB pathway is involved in regulating mitochondrial function. Incubation of primary Schwann cells with NRG1 for 24 hours stimulates mitochondrial biogenesis and increases mitochondrial density through the extracellular signal-related kinase (ERK) and the phosphoinositide 3-kinase (PI3K) signalling pathways^11^. In a rat model of heart failure, treatment with NRG1 for 10 days restored mitochondrial respiration rate, mitochondrial membrane potential and adenosine triphosphate (ATP) concentrations compared with untreated controls^12^. In agreement, when primary neonatal rat ventricular myocytes are cultured in the presence of an anti-ERBB2 antibody, they display mitochondrial dysfunction, loss of mitochondrial membrane potential, reduced ATP levels and loss of redox capacity caused by activation of the mitochondrial apoptosis pathway^13^. Similar effects are often observed in the heart in response to anticancer therapies that target ERBB2^14^. These findings clearly implicate NRG1/ERBB signalling in the regulation of heart mitochondrial function *in vivo*. On the other hand, only one *in vitro* study has been performed in skeletal muscle cells^10^, but many data concerning other tissues or cellular models suggest that the NRG1/ERBB pathway could be crucial for the regulation of mitochondrial oxidative capacity in skeletal muscle as well. However, NRG1 effect on skeletal muscle mitochondrial function has never been addressed *in vivo*.

Moreover, NRG1/ERBB abundance and signalling are defective in many tissues in different models of diabetes^15–21^. In particular, diabetic mice exhibit a significant decrease in ErbB2 and ErbB4 mRNA expression and NRG1 protein abundance in the left ventricular myocardium^19^. In the liver, hyperinsulinemia has been shown to downregulate ErbB3 receptors^15^, while ErbB2 signalling was altered in sciatic nerve of diabetic mice^18^. As impaired mitochondrial function could contribute to the pathogenesis of insulin resistance in skeletal muscle^22^, we investigated whether chronic treatment with NRG1 improves mitochondrial function in mouse skeletal muscle. We also tested the hypothesis that NRG1/ERBB abundance and signalling are disturbed in skeletal muscle of db/db mice and that chronic treatment with NRG1 improves mitochondrial function in this animal model of type 2 diabetes.

## Results

### Weight, body composition and food intake

As expected, diabetic db/db mice ate more than control mice, with higher global caloric intake over the 8 weeks of experiments (1078 ± 135 kcal vs 743 ± 41 kcal respectively; p < 0.001). Diabetic db/db mice presented a higher body weight than control mice both at baseline (43.3 ± 2.7 g vs 26.3 ± 1.9 g; p < 0.001) and at the end of the treatment (40.0 ± 4.9 g vs 26.8 ± 1.5 g; p < 0.001). Similarly, body fat mass was higher in diabetic db/db mice compared with control mice both at baseline (22.6 ± 2.7 g vs 3.4 ± 0.6 g; p < 0.001) and at the end of the treatment (16.8 ± 4.9 g vs 3.3 ± 0.7 g; p < 0.001). Finally, NRG1 treatment for 8 weeks slightly reduced body weight gain compared with untreated mice (−1.4 ± 2.7 g vs 0.2 ± 2.1 g; p < 0.05).

### Mitochondrial biogenesis and function

**Figure 1:**
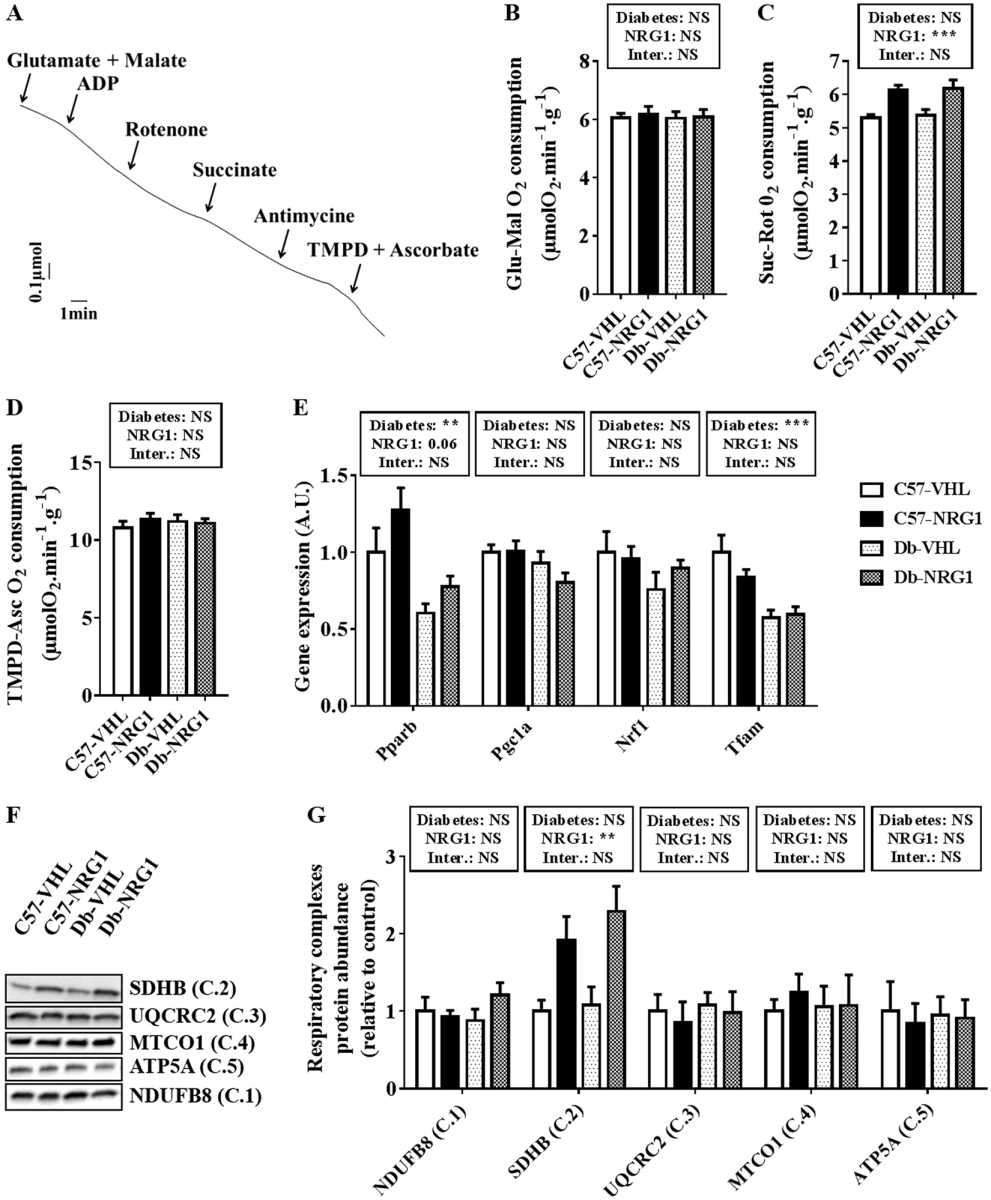
NRG1 treatment improves complex 2-mediated mitochondrial respiration in *gastrocnemius* muscle. C57BL/6JRJ (C57) and db/db (Db) male mice were treated with vehicle (VHL; 0.9% NaCl solution; n = 8/condition) or with NRG1 (50 μg . kg^−1^; n = 8/condition), three days per week for eight weeks. Representative recording (**A**) of ADP-stimulated maximal mitochondrial O_2_ consumption assessed in permeabilised *gastrocnemius* muscle fibres with glutamate and malate (Glu-Mal) (**B**), succinate and rotenone (Suc-Rot) (**C**) or TMPD and ascorbate (TMPD-Asc) (**D**) as substrates and inhibitors. The expression of genes involved in mitochondrial biogenesis (*Pparb*, *Pgc1a*, *Nrf1* and *Tfam*) was assessed by RT-qPCR (**E**). The abundance of respiratory complex subunits was analysed by western blotting ((**F**) cropped images). Quantification of the abundance of respiratory complexes subunits (**G**) was relative to the level in untreated healthy mice (C57-VHL, white bars). Gene expression levels were calculated using the absolute quantification method and a cDNA calibration curve, and it is shown as relative change of the expression in untreated healthy mice (C57-VHL, white bars). Results are the mean ± SEM, (n = 8 per group). The diabetes (healthy vs db/db mice) and NRG1 (saline vs NRG1) effects were investigated with a 2 × 2 ANOVA. When a significant interaction was found, the Tuckey’s test was used for post-hoc multiple comparisons. **p < 0.01, ***p < 0.001, NS: not significant.

The mitochondrial maximal oxygen consumption rate was comparable in *gastrocnemius* bundles from untreated (VHL) db/db (Db) and healthy control (C57) mice, whatever the substrate (Fig. 1A–D). Although the expression of the genes encoding PPARβ (p < 0.01, Fig. 1E) and TFAM (involved in mitochondrial transcription regulation) (p < 0.001, Fig. 1E) was significantly reduced in untreated db/db mice compared with controls, no change was observed in the protein abundance of porin (a mitochondrial membrane protein, Supplementary Fig. S1) and of components of the respiratory chain complexes (Fig. 1G). Compared with untreated mice (VHL), chronic treatment with NRG1 induced a significant increase in the ADP-stimulated maximal oxygen consumption rate (about 15%) in both healthy and diabetic mice, but only in the presence of succinate and rotenone (Suc-Rot), a specific substrate of the respiratory chain complex 2 (Fig. 1C, p < 0.001). This result was corroborated by the 2-fold increase in the abundance of the mitochondrial complex 2 subunit succinate dehydrogenase iron-sulphur subunit (SDHB) upon NRG1 treatment in both db/db and healthy mice (Fig. 1G, p < 0.01). Conversely, the protein levels of porin and the other respiratory chain complexes were not modified by NRG1. Similarly, NRG1 treatment did not significantly affect the expression of genes involved in mitochondrial biogenesis, although *Pparb* level was slightly increased in treated compared with untreated *gastrocnemius* samples (p = 0.06, Fig. 1E).

### Regulators of muscle energy metabolism

**Figure 2:**
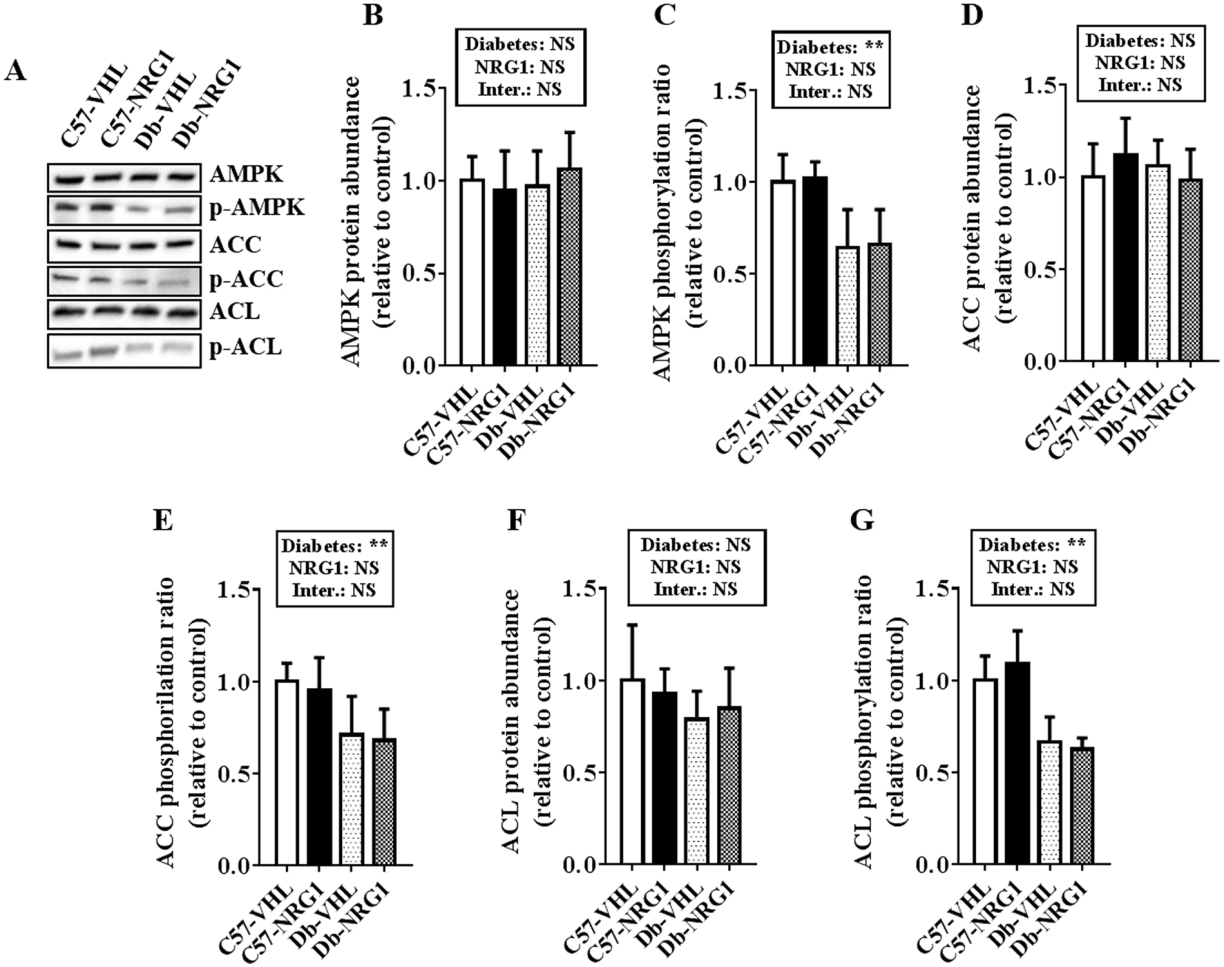
NRG1 treatment does not affect the main regulators of energy homeostasis in *gastrocnemius* muscle. C57BL/6JRJ (C57) control and db/db (Db) male mice were treated with vehicle (VHL; 0.9% NaCl solution; n = 8/each condition) or with NRG1 (50 μg . kg^−1^; n = 8/each condition), three days per week for eight weeks. Western blot analysis ((**A**) cropped images) was used to quantify the abundance of AMPK (**B**), ACC (**D**), ACL (**F**) and their phosphorylation ratios (**C**,**E** and **G**) relative to the level in untreated healthy mice (C57-VHL, white bars). Results are the mean ± SEM (n = 8 per group). The diabetes (healthy vs db/db mice) and NRG1 (saline vs NRG1) effects were investigated with a 2 × 2 ANOVA. When a significant interaction was found, the Tuckey’s test was used for post-hoc multiple comparisons. **p < 0.01, NS: not significant.

Previous studies showed that NRG1 increases the abundance of the insulin-regulated glucose transporter type 4 (GLUT4) and stimulates fatty acid oxidation in skeletal muscle cells^10^. Assessment of the abundance of factors involved in fatty acid oxidation (5′ AMP-activated protein kinase, AMPK; acetyl-CoA carboxylase, ACC; ATP citrate lyase, ACL) showed that, the phosphorylation ratios of AMPK (Fig. 2C, p < 0.01), ACC (Fig. 2E, p < 0.01) and ACL (Fig. 2G, p < 0.01) were reduced in untreated (VHL) db/db (Db) mice compared with control (C57) mice. NRG1 treatment did not modify AMPK, ACC and ACL protein levels and phosphorylation ratios (Fig. 2A–G). GLUT4 protein abundance (Supplementary Fig. S2) and the mRNA levels of genes involved in lipid metabolism (*Cd36*, *Cpt1a* and *b*, and *Acadm;* Supplementary Fig. S3) were not significantly different in diabetic and healthy mice both before and after NRG1 treatment.

### NRG1/ERBB signalling

**Figure 3:**
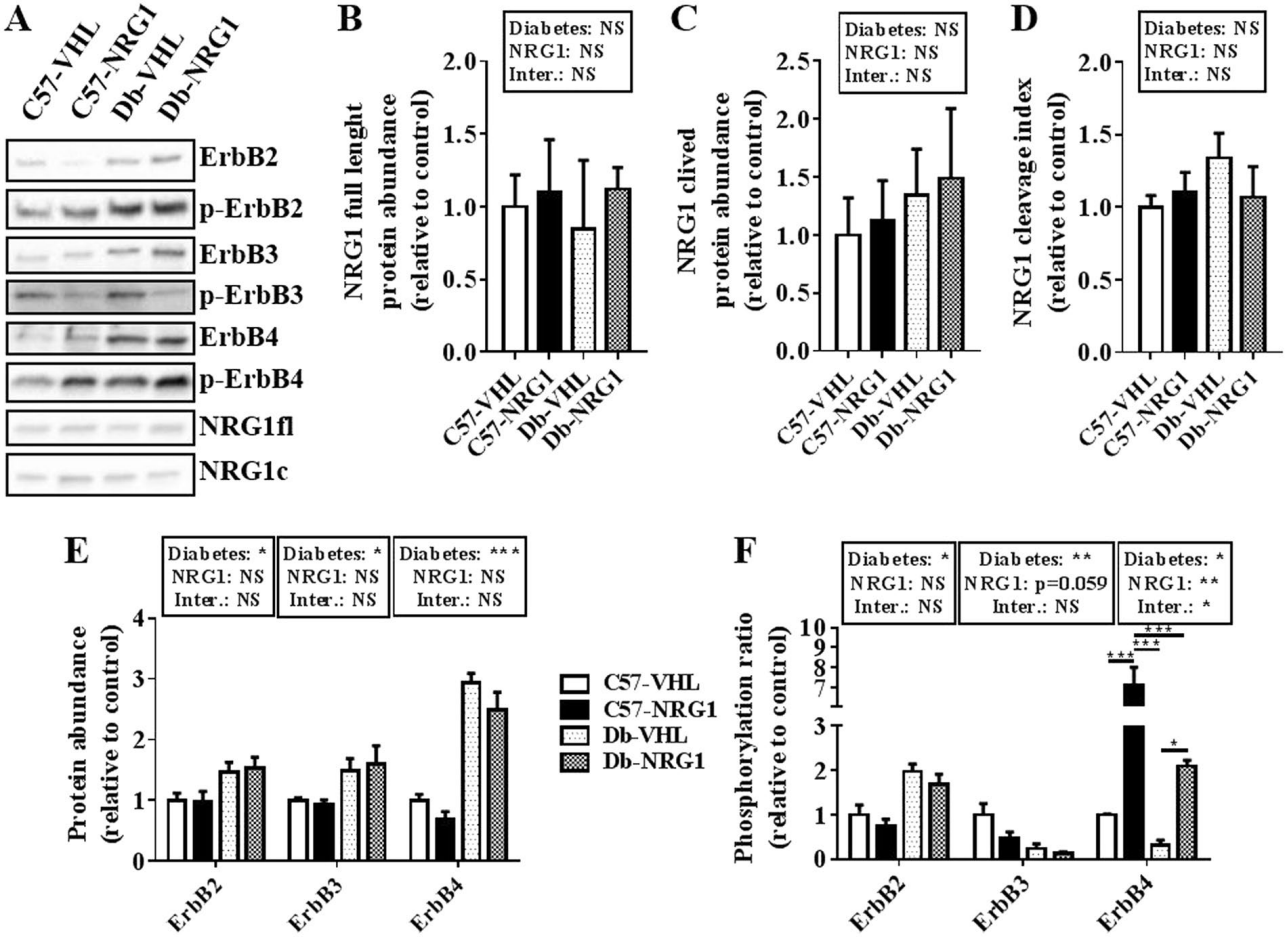
NRG1 treatment increases ERBB4 activation in *gastrocnemius* muscle. C57BL/6JRJ (C57) control and db/db (Db) male mice were treated with vehicle (VHL; 0.9% NaCl solution; n = 8/each condition), or with NRG1 (50 μg . kg^−1^; n = 8/each condition), three days per week for eight weeks. Western blot analysis (**A**) cropped images) was used to quantify in *gastrocnemius* muscle samples the abundance of full length (115 kDa) (**B**) and cleaved (42 kDa) (**C**) NRG1 and the NRG1 cleavage index (the ratio between cleaved and full length NRG1) (**D**) as well as the abundance (**E**) and phosphorylation ratios (**F**) of ERBB2, ERBB3 and ERBB4. Results are the mean ± SEM (n = 8 per group) relative to the level in untreated healthy mice (C57-VHL, white bars). Diabetes (healthy vs db/db mice) and NRG1 (saline vs NRG1) effects were investigated with a 2 × 2 ANOVA. When a significant interaction was found, the Tuckey’s test was used for post-hoc multiple comparisons. *p < 0.05, **p < 0.01, ***p < 0.001, NS: not significant.

Finally, we quantified the abundance of full length (inactive) and cleaved (active) NRG1 as well as the abundance and phosphorylation ratio of the ERBB receptors and of AKT^23^ and ERK1/2, the two main pathways involved in NRG1/ERBB signalling^24^. NRG1 protein levels were not significantly different in untreated (VHL) and treated (NGR1) healthy (C57) and diabetic (Db) animals (Fig. 3B–D). Conversely, abundance of ERBB2 (p < 0.05), ERBB3 (p < 0.05) and ERBB4 (p < 0.001) was significantly higher in untreated db/db mice than healthy controls (Fig. 3E). Quantification of the ERBB phosphorylation ratios, to monitor their activation, showed that ERBB2 phosphorylation was increased (p < 0.05), whereas ERBB3 (p < 0.01) and ERBB4 (p < 0.05) phosphorylation were reduced in untreated db/db mice compared with healthy controls (Fig. 3F). Chronic treatment with NRG1 did not modify ERBB abundance in both groups (Fig. 3E). Conversely, it slightly reduced ERBB3 phosphorylation ratio (p = 0.059) and induced a marked and significantly increase in ERBB4 phosphorylation ratio (p < 0.01) in both healthy and db/db animals (Fig. 3F). Post-hoc multi-group comparisons indicated that ERBB4 phosphorylation ratio was higher in the treated healthy group than in the other three groups (p < 0.001), and in treated than in untreated db/db mice (p < 0.05) (Fig. 3F). AKT (Supplementary Fig. S4) and ERK1/2 (Supplementary Fig. S5) protein levels and phosphorylation ratios were not different in untreated and treated db/db and control mice.

## Discussion

In this study, we show that chronic NRG treatment improves complex 2-mediated mitochondrial respiration in the *gastrocnemius* of healthy and db/db diabetic mice. This effect is not associated with an increase in the expression of mitochondrial biogenesis markers. NRG1 effects on mitochondrial function could be mediated through enhanced ERBB4 phosphorylation and activation of signalling pathways, independently of ERK1/2, AKT and AMPK.

To our knowledge, this is the first study on NRG1/ERBB signalling in skeletal muscle in diabetic mice. We previously showed that NRG1 and ERBB abundance and activation are not modified in Wistar rats with high fat diet-induced obesity compared with lean controls^8^. Here, we found that ERBB2, 3 and 4 protein levels are higher in skeletal muscle of db/db mice than healthy controls. Similarly, the phosphorylation ratio of ERBB2 is higher and that of ERBB3 and ERBB4 is lower in mice with diabetes compared with controls. This suggests that the metabolic alteration in db/db mice affects ERBB expression and activation. The altered ERBB phosphorylation state in db/db mice does not seem to be related to modifications of NRG1 abundance or cleavage, which are not different between diabetic and healthy controls. However, other ERBB ligands could be involved in regulating ERBB activation in db/db mice. Indeed, other ligands, which have been involved in metabolic disorders, can modulate ERBB phosphorylation state^25^, such as NRG4^26, 27^, betacellulin^28, 29^, epiregulin^30^ or proheparin-binding EGF-like growth factor (HB-EGF)^31, 32^. As many biological processes can be regulated through ERBB, the functional consequences of the modified ERBB abundance and phosphorylation state in diabetes are difficult to predict. Our results indicate that the abundance and activation of the two main signalling molecules regulated by ERBB activation (AKT and ERK1/2) are not affected in db/db mice. Nevertheless, we cannot rule out the possibility that other signalling pathways might be altered. Indeed, phosphorylation of AMPK and ACC, two powerful regulators of muscle energy metabolism^33^, also was reduced in db/db mice compared with healthy controls, as previously reported^34, 35^. ACL phosphorylation also was reduced in skeletal muscle of diabetic mice. As ACL is considered to be an important regulator of mitochondrial function^36^, we could hypothesise that ACL dysregulation might contribute to the disturbed oxidative muscle metabolism associated with diabetes^37^. However, we did not find any significant impairment of mitochondrial biogenesis and function in db/db mice. Despite a reduction in *Pparb* and *Tfam* gene expression, the abundance of porin and of subunits of the respiratory chain complexes were not modified in *gastrocnemius* samples from db/db mice. Similarly, mitochondrial O_2_ consumption, assessed in permeabilised fibres, was comparable in diabetic db/db and healthy animals. Previous studies on the mitochondrial function in skeletal muscle of db/db mice have reported conflicting results. Increased oxidative enzyme activities and PGC1α protein content have been observed in the *quadriceps* of 12-week-old male db/db mice^38^. Conversely, citrate synthase activity and mitochondrial DNA content were not changed in the *biceps femoris* of 6-month-old male db/db mice^39^. In this last study, mitochondrial respiration in isolated mitochondria from *tibialis anterior* and *gastrocnemius* muscles was not altered by the diabetic condition. In *soleus* and *gastrocnemius* muscles of 9 week-old male db/db mice, mitochondrial DNA was decreased, but the activities of oxidative enzymes were unchanged or increased^40^. Finally, in 16- to 19-week-old db/db mice, mitochondrial respiration was increased in the *extensor digitorum longus* muscle (fast-type, glycolytic), but decreased in the *soleus* muscle (slow-type, oxidative). This suggests differential effects of diabetes on muscle mitochondrial function, depending on the muscle fibre type profile^41^. Here, we evaluated mitochondrial function in the *gastrocnemius*, a muscle with mixed fibre type profile, albeit with more glycolytic fibres^42^. In agreement with the literature, we found almost no impairment of mitochondrial function in the *gastrocnemius* of db/db mice. We also cannot rule out the possibility that studying db/db mice at a later diabetic stage (*i.e*. in older mice), would have highlighted mitochondrial dysfunctions. Indeed, several studies revealed that mitochondrial content is unchanged or even sometimes increased at the early stage of insulin resistance and diabetes in various rodent models^38, 43, 44^. In the present study, the absence of modifications in the mitochondrial protein level and respiration suggests that the decreased phosphorylation (activation) of AMPK, ACC and ACL is compensated through unknown mechanisms, or that the observed reduction is not enough to induce functional changes in mitochondria.

In both db/db and healthy mice, skeletal muscle NRG1 protein level (full length or cleaved) was not modified following chronic NRG1 treatment, suggesting that the negative feedback regulation on NRG1 expression does not take place in treated mice. Similarly, ERBB abundance were not affected by NRG1 treatment, whereas ERBB4 phosphorylation ratio was markedly increased (by around 7-fold) compared with untreated controls. The functional consequences of ERBB4 phosphorylation increase remains unclear. Recently, Pentassuglia and colleagues showed that NRG1 phosphorylates (activates) AKT at Ser473 and Thr308 in neonatal rat cardiomyocytes^45^. Here, chronic NRG1 treatment did not alter basal and phosphorylated (Ser473 and Thr308) AKT level. These results are in accordance with the study by Lopez-Soldadό and colleagues who showed that acute injection of NRG1 does not activate AKT in skeletal muscle of lean and diabetic Zucker rats^46^. The other molecules evaluated (ERK1/2, AMPK, ACC and ACL) also were not affected by NRG1 treatment. Further studies are needed to identify the downstream signalling pathways that are regulated by ERBB4 activation in skeletal muscle. It has been shown that ERBB4 intracellular domain has numerous tyrosine residues and interaction partners^47, 48^. We could thus hypothesise that NRG1 may activate other downstream signalling pathways mediating the effects on mitochondria. Also, we cannot rule out the hypothesis that the timing of the present study has hidden the NRG1-induced transient activation of some signalling pathways downstream ErbB4. Indeed, in order to distinguish between the acute and chronic effects of NRG1 treatment, we chose to sacrifice the mice 3 days after the last NRG1 injection. Nevertheless, circulating half-life of NRG1 is quite short (approximately 30 min)^49^. *In vivo*, NRG1-induced ERK1/2 activation in the heart is maximal at 90 min post-injection and decreases thereafter^49^. We can thus hypothesise that each NRG1 injection could transiently activate some signalling pathways that may account for the chronic effects of the treatment.

Indeed, we also showed that chronic treatment with NRG1 improved complex 2-mediated mitochondrial respiration in *gastrocnemius* muscles in both diabetic and healthy animals. Studies assessing NRG1 effect on oxidative capacity are scarce. This is the first *in vivo* study that investigated the effect of chronic NRG1 treatment on mitochondrial function in skeletal muscle. We observed a significant effect of NRG1 treatment on the electron flow through complex 2, in agreement with the significant increase in complex 2 subunit content. Cantό and colleagues found that chronic NRG1 treatment improves mitochondrial oxidative capacity in L6E9 muscle cells^10^. They demonstrated that NRG1 increases mitochondrial protein content (porin and all respiratory chain subunits) through a mechanism involving PPARβ. Here, we only observed an increase of PPARβ mRNA level, whereas the other markers of mitochondrial biogenesis and density remained unchanged. These results are consistent with a specific effect of NRG1 treatment on complex 2 subunit expression and functionality, rather than a global stimulation of mitochondrial biogenesis. Altogether, these findings show that NRG1 can regulate mitochondrial function. Further studies are needed to understand the precise mechanisms involved in NRG1-induced improvement in complex 2-mediated mitochondrial respiration in skeletal muscle. Moreover, we can also hypothesise that NRG1 treatment could affect mitochondrial production of reactive oxygen species (ROS). Indeed, NRG1 was shown to attenuate oxidative stress in microglial and cardiac cells^50, 51^. It is also well accepted that increased mitochondrial ROS production is associated with insulin resistance and diabetes^52^. Moreover, excess of ROS production may promote calcium homeostasis impairment leading to contractility defect^53–55^. Studies dealing with the effects of NRG1 treatment on mitochondrial ROS production in skeletal muscle would be thus of interest.

**Figure 4:**
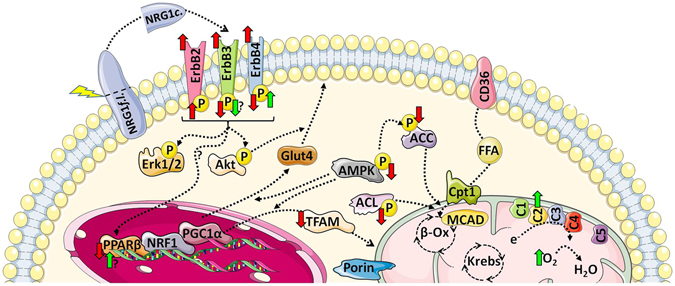
Summary of the main effects of diabetes and NRG1 treatment in *gastrocnemius* muscle. The abundance of ERBB receptors is increased in diabetic db/db mice. This is associated with increased phosphorylation ratio of ERBB2 and decreased phosphorylation ratios of ERBB3 and ERBB4. Similarly, phosphorylation (activation) of the metabolic regulators AMPK, ACC and ACL is reduced in diabetic mice as well as *Pparb* and *Tfam* mRNA expression level. However, mitochondrial respiration in permeabilised fibres is similar in diabetic and control healthy mice. Chronic NRG1 treatment increases ERBB4 phosphorylation and tends to decrease ERBB3 phosphorylation. Among the regulators of the mitochondrial biogenesis pathway, only *Pparb* mRNA expression is slightly increased by NRG1. However, in NRG1-treated mice, complex 2-mediated mitochondrial respiration and complex 2 subunit abundance are increased. Effect of diabetes: red arrows. Effect of NRG1: green arrows. Figure was produced using *Servier Medical Art* image bank (http://www.servier.com/Powerpoint-image-bank), which is licensed under a Creative Commons Attribution 3.0 Unported License (http://creativecommons.org/licenses/by/3.0/).

In summary (Fig. 4), we found that in db/db mice, ERBB abundance and phosphorylation state are altered and that the phosphorylation ratios of key enzymes involved in energy metabolism regulation (AMPK, ACC and ACL) are decreased. This is not accompanied by changes in mitochondrial density and function. Chronic NRG1 treatment improved complex 2-mediated mitochondrial respiration, possibly through upregulation of complex 2 subunit expression. ERBB4 could mediate NRG1 effects on mitochondria in skeletal muscle, but the downstream signalling pathways remain to be elucidated. As a previous study reported that chronic NRG1 treatment improves glucose tolerance in db/db mice^56^, targeting the NRG1 pathway may represent a promising therapeutic strategy in conditions of insulin resistance, especially when a clear defect on mitochondrial respiration on complex 2 is underlined.

## Materials and Methods

### Animals

Animal husbandry and experimental procedures were in accordance with the EU Directive 2010/63/EU for animal experiments and were approved by the local ethics committee for animal experimentation (CEMEA Auvergne, CE1-09). Eight-week-old C57BL/6JRJ (healthy controls) and BKS(D)-*Lepr* ^*db*^ */*JOrlRj (db/db) male mice were obtained from CERJ Janvier (Le Genest Saint Isle, France). Each mouse was kept in a temperature-controlled cage (20–22 °C) with a reversed light-dark cycle (20 h00-8 h00) and had free access to water and food (standard A03 chow diet, Safe, Augy, France).

### Experimental design and NRG1 treatment

Healthy and db/db mice were randomly divided in two groups. The first group received an *i.p*. injection of 50 µg . kg^−1^ body weight of recombinant human NRG1 (Zensun Sci & Tech Ltd, Shanghai, China), 3 times per week for eight weeks (NRG1 group, n = 8/each condition). The second group received an equal volume of 0.9% NaCl (untreated group, n = 8/each condition). Treatment was stopped three days before euthanasia.

### Animal dissection

Mice were anesthetised with isoflurane and euthanised by decapitation after 6 h of fasting. The *gastrocnemius* muscles were dissected, weighed, and isolated for direct mitochondrial oxygen consumption assessment or frozen in liquid nitrogen and stored at −80 °C for later biochemical analysis.

### Mitochondrial oxygen consumption

About 50 mg of wet tissue (*gastrocnemius*) was used for isolation of saponin-permeabilised fibres. Bundles of muscle fibres were isolated by mechanical dissection in ice-cold relaxing solution (10 mM EGTA–calcium buffer: 100 nM free Ca^2+^, 20 mM imidazole, 3 mM KH_2_PO_4_, 1 mM MgCl_2_, 20 mM Taurine, 0.5 mM DTT, 5 mM MgATP and 15 mM Phosphocreatine, pH 7.1). After incubation with 5 mg.ml^−1^ saponin in relaxing solution at 4 °C for 30 min, muscle bundles were rinsed and placed in a respiration chamber at 27 °C in respiratory medium (130 mM KCl, 2 mM KH_2_PO_4_, 3 mM HEPES, 2 mM MgCl_2_, 1 mM EGTA, 2 mM fatty acid-free bovine serum albumin, BSA). The ADP-stimulated (2 mM) maximal respiratory rate was recorded with a Clark electrode (Strathkelvin, Glasgow, Scotland) in the presence of 5 mM glutamate and 2 mM malate, 10 mM succinate and 20 μM rotenone, or 5 μM TMPD, 2 mM ascorbate and 6.5 μM antimycin.

### Protein extraction

*Gastrocnemius* muscle tissue (50 mg) was homogenised in 400 µl lysis buffer (20 mM Hepes, 350 mM NaCl, 20% (v/v) glycerol, 1% (v/v) Nonidet P-40, 1 mM MgCl_2_, 0.5 mM EDTA, 0.1 mM EGTA, pH 7.9) supplemented with a protease inhibitor cocktail (Sigma-Aldrich Co.), using a Potter-Elvehjem tissue grinder placed on ice. Homogenates were centrifuged at 10 000 g for 5 min and supernatants stored at −80 °C for further analysis. Protein content was measured by using the Bradford method (Biorad Laboratories) and BSA as standard (Sigma-Aldrich Co.). Homogenates were diluted with lysis buffer to a final concentration of 30 µg . ml^−1^.

### Western blotting

Protein samples were diluted with Laemmli buffer, separated on Criterion Stain-Free precast gels in a Mini PROTEAN Tetra-Cell unit (BioRad, USA) and transferred to nitrocellulose membranes using a Trans Blot Turbo transfer (BioRad, USA). Membranes were blocked with 5% non-fat dry milk in Tris buffered saline (pH 7.5) containing 0.1% Tween 20 (TBST) at room temperature for 1 h. Then, membranes were incubated in 2% BSA with the relevant primary antibodies at 4 °C overnight. The anti-OXPHOS (1/1000) antibody was from Abcam (Cambridge, UK). The anti-porin (1/1000), -AKT (1/1000), -p-AKT Ser473 (1/1000), -p-AKT Thr308 (1/1000), -ERK (1/1000), -p-ERK, (1/1000), -AMPK (1/1000), -p-AMPK (1/1000), ACC (1/1000), -p-ACC (1/1000), -ACL (1/1000), -p-ACL (1/1000), -GLUT4 (1/1000), -ERBB3 (1/200) and -p-ERBB3 (1/200) antibodies were purchased from Cell Signaling (Beverly, MA, USA). The anti-ERBB2 (1/200), -ERBB4 (1/200), -p-ERBB2 (1/200), -p-ERBB4 (1/200) and -NRG1 (1/1000) antibodies were purchased from Santa Cruz (Santa Cruz Biotechnology, CA, USA). After incubation with the appropriate horseradish peroxidase-conjugated secondary antibody in TBST at room temperature for 1 h, enhanced chemiluminescence (ECL) reagents (BioRad, USA) were used to detect interactions and digital images were acquired using the Molecular Imager ChemiDoc XRS System (Biorad, USA). Signals were quantified using the Image Lab 4.1 software (BioRad, USA) and normalised using the Total Protein Normalization (TPN) method provided by Stain-Free Technology^57^.

### Real-time Quantitative Polymerase Chain Reaction (RT-qPCR)

Total RNA was extracted from frozen *gastrocnemius* muscle tissue samples using the TRIzol reagent (Invitrogen, Carlsbad, CA) following the manufacturer’s instructions. The integrity of all RNA samples was confirmed by gel electrophoresis. First strand cDNA was synthesised using the High Capacity cDNA Reverse Transcription Kit (Applied Biosystem, USA) and expression of the genes encoding PPARβ, PGC-1α, TFAM, NRF1, CPT1a, CPT1b, CD36 and MCAD was assessed by qPCR on a Rotor Gene cycler (Qiagen, Courtaboeuf, France) using the Rotor-Gene SYBR Green PCR Kit (Qiagen). Gene expression levels were calculated using the absolute quantification method and a cDNA calibration curve.

### Statistical analysis

Data are presented as the mean ± SEM. The pathology (healthy vs db/db mice) and treatment (saline vs NRG1) effects were investigated with a 2 × 2 ANOVA. When a significant effect was found, the Tuckey’s post-hoc test was used for post-hoc multiple comparisons. Statistical significance was set at 0.05.

## Electronic supplementary material

Supplementary Information
