# Supplementary material for: Neuregulin 1 improves complex 2-mediated mitochondrial respiration in skeletal muscle of healthy and diabetic mice
Source: Sci Rep. 2017 May 11;7:1742. doi: 10.1038/s41598-017-02029-z (PMC5431817; doi:10.1038/s41598-017-02029-z)
Supplement: Supplementary file 1 — Supplementary Information [file 41598_2017_2029_MOESM1_ESM.pdf]

Supplementary Information

Neuregulin 1 improves complex 2-mediated mitochondrial respiration in skeletal muscle of healthy and diabetic mice

Gaël Ennequin[a,d,*], Frederic Capel[b], Kevin Caillaud[a], Vivien Chavanelle[a], Monique Etienne[a], Allison Teixeira[a], Xinyan Li[c], Nathalie Boisseau[a], Pascal Sirvent[a]

[a]Université Clermont Auvergne, Laboratoire des Adaptations Métaboliques à l’Exercice en Conditions Physiologiques et Pathologiques (AME2P), F-63000 Clermont-Ferrand, France.
[b]INRA UMR1019 Nutrition Humaine, Laboratoire de Nutrition Humaine, Université d'Auvergne, CRNH, 58 rue Montalembert BP321, 63009 Clermont Ferrand CEDEX 1, France.
[c]Zensun Sci & Tech Ltd., Shanghai, China
[d]PEPITE EA4267 and Exercise Performance Health Innovation Platform Univ. Bourgogne Franche-Comté, F-25000 Besançon, France

*Corresponding author gael.ennequin@univ-fcomte.fr

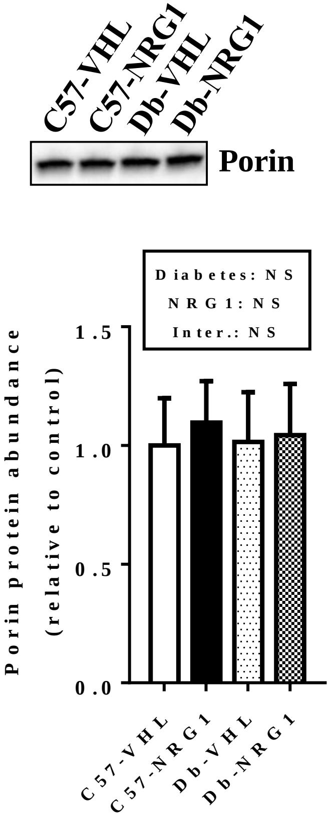

Supplementary Figure S1. The abundance of porin was analyzed by western blotting. Representative cropped image of western blot is shown. Quantification was relative to the level in untreated healthy mice (C57-VHL, white bars). Results are the mean ± SEM, (n = 8 per group). The diabetes (healthy vs db/db mice) and NRG1 (saline vs NRG1) effects were investigated with a 2 x 2 ANOVA. NS: not significant.

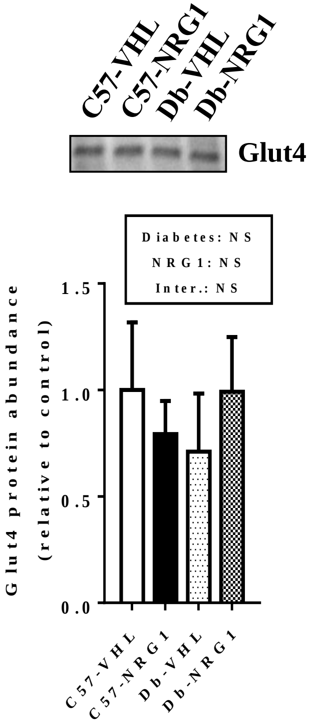

Supplementary Figure S2. The abundance of GLUT4 was analyzed by western blotting. Representative cropped image of western blot is shown. Quantification was relative to the level in untreated healthy mice (C57-VHL, white bars). Results are the mean ± SEM, (n = 8 per group). The diabetes (healthy vs db/db mice) and NRG1 (saline vs NRG1) effects were investigated with a 2 x 2 ANOVA. NS: not significant.

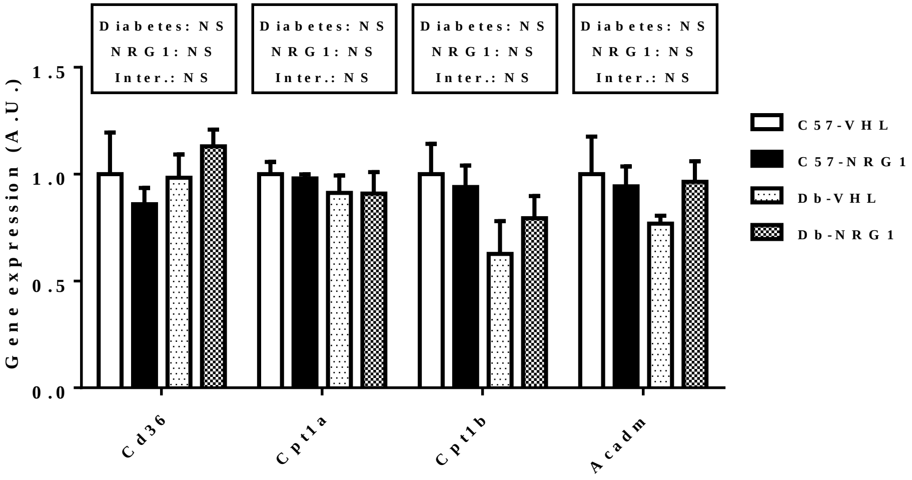

Supplementary Figure S3. Genes involved in the lipid oxidation pathway (*Cd36*, *Cpt1a*, *Cpt1b* and *Acadm*) were assessed by RT-qPCR Gene expression levels were calculated using the absolute quantification method and a cDNA calibration curve, and it is shown as relative change of the expression in untreated healthy mice (C57-VHL, white bars). Results are the mean ± SEM, (n = 8 per group). The diabetes (healthy vs db/db mice) and NRG1 (saline vs NRG1) effects were investigated with a 2 x 2 ANOVA. NS: not significant.

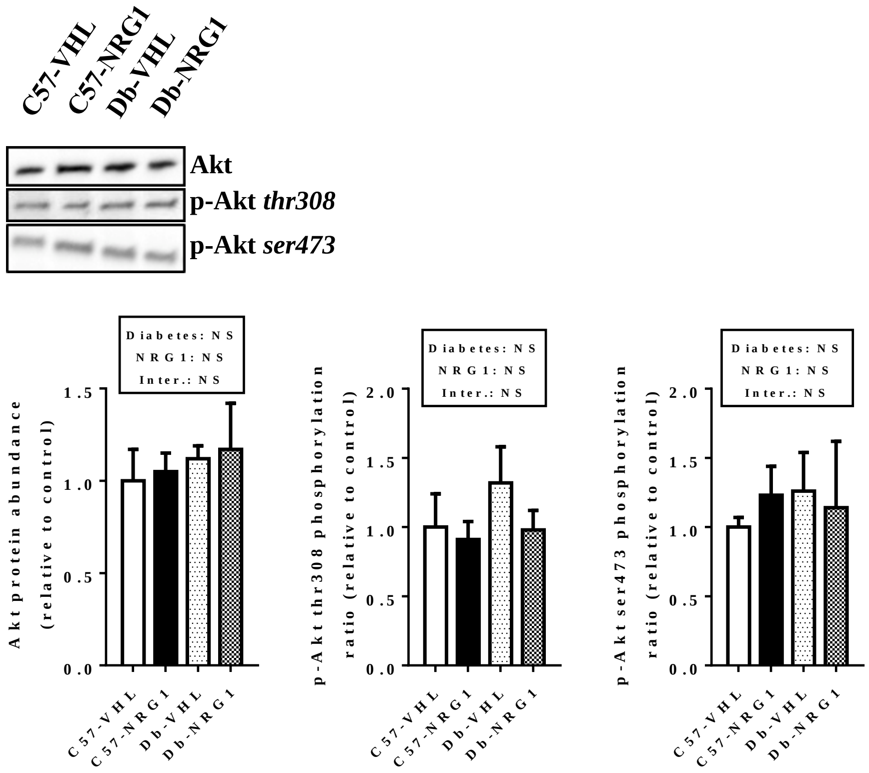

Supplementary Figure S4. The abundance and phosphorylation ratios of Akt were analyzed by western blotting. Representative cropped image of western blot is shown. Quantification was relative to the level in untreated healthy mice (C57-VHL, white bars). Results are the mean ± SEM, (n = 8 per group). The diabetes (healthy vs db/db mice) and NRG1 (saline vs NRG1) effects were investigated with a 2 x 2 ANOVA. NS: not significant.

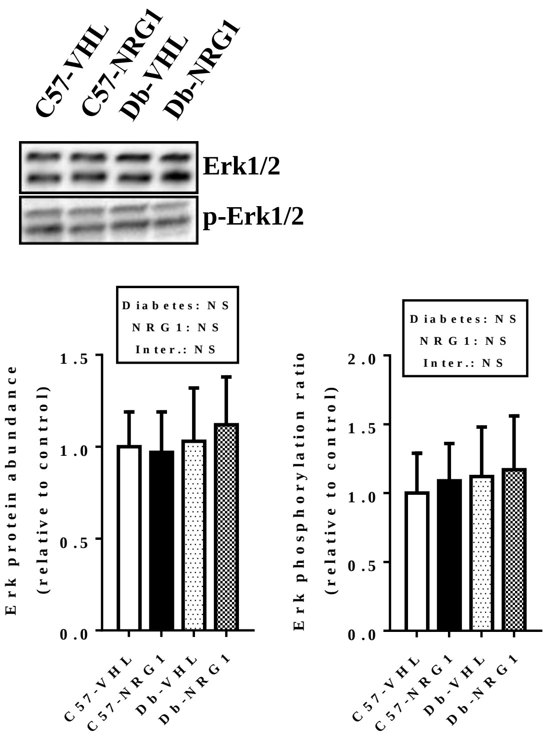

Supplementary Figure S5. The abundance and phosphorylation ratios of Erk1/2 were analyzed by western blotting. Representative cropped image of western blot is shown. Quantification was relative to the level in untreated healthy mice (C57-VHL, white bars). Results are the mean ± SEM, (n = 8 per group). The diabetes (healthy vs db/db mice) and NRG1 (saline vs NRG1) effects were investigated with a 2 x 2 ANOVA. NS: not significant.
